# Chemotherapy Regimens Received by Women With *BRCA1/2* Pathogenic Variants for Early Stage Breast Cancer Treatment

**DOI:** 10.1093/jncics/pkac045

**Published:** 2022-06-20

**Authors:** Allison W Kurian, Paul Abrahamse, Ann S Hamilton, Jennifer L Caswell-Jin, Scarlett L Gomez, Timothy J Hofer, Kevin C Ward, Steven J Katz

**Affiliations:** Department of Medicine, Stanford University, Stanford, CA, USA; Department of Epidemiology and Population Health, Stanford University, Stanford, CA, USA; Department of Health Management and Policy, School of Public Health and Department of Internal Medicine, University of Michigan, Ann Arbor, MI, USA; Department of Population and Public Health Sciences, Keck School of Medicine, University of Southern California, Los Angeles, CA, USA; Department of Medicine, Stanford University, Stanford, CA, USA; Department of Epidemiology & Biostatistics and Hellen Diller Family Comprehensive Cancer Center, University of California San Francisco, San Francisco, CA, USA; Department of Internal Medicine, University of Michigan and Center for Clinical Management Research, Veterans Affairs Ann Arbor Healthcare System, Ann Arbor, MI, USA; Department of Epidemiology, Rollins School of Public Health, Emory University, Atlanta, GA, USA; Department of Health Management and Policy, School of Public Health and Department of Internal Medicine, University of Michigan, Ann Arbor, MI, USA

## Abstract

**Background:**

Genetic testing is widespread among breast cancer patients; however, no guideline recommends using germline genetic testing results to select a chemotherapy regimen. It is unknown whether breast cancer patients who carry pathogenic variants (PVs) in *BRCA1* and/or *2* (*BRCA1/2*) or other cancer-associated genes receive different chemotherapy regimens than noncarriers.

**Methods:**

We linked Surveillance, Epidemiology, and End Results registry records from Georgia and California to germline genetic testing results from 4 clinical laboratories. Patients who 1) had stages I-III breast cancer, either hormone receptor (HR) positive and HER2 negative or triple negative (TNBC), diagnosed in 2013-2017; 2) received chemotherapy; and 3) were linked to genetic results were included. Chemotherapy details were extracted from Surveillance, Epidemiology, and End Results text fields completed by registrars. We examined whether PV carriers received more intensive regimens (HR-positive,HER2-negative: ≥3 drugs including an anthracycline; TNBC: ≥4 drugs including an anthracycline and platinum) and/or less standard breast cancer agents (a platinum). All statistical tests were 2-sided.

**Results:**

Among 2293 patients, 1451 had HR-positive, HER2-negative disease, and 842 had TNBC. On multivariable analysis of women with HR-positive, HER2-negative disease, receipt of a more intensive chemotherapy regimen varied statistically significantly by genetic results (*P* = .02), with platinum receipt more common among *BRCA1/2* PV carriers (odds ratio = 2.44, 95% confidence interval = 1.36 to 4.38; *P* < .001). Among women with TNBC, chemotherapy agents did not vary significantly by genetic results.

**Conclusion:**

*BRCA1/2* PV carriers with HR-positive, HER2-negative breast cancer had twofold higher odds than noncarriers of receiving a platinum, as part of a more intensive chemotherapy regimen. This likely represents overtreatment and emphasizes the need to monitor how genetic testing results are managed in oncology practice.

Germline genetic testing is widespread after a breast cancer diagnosis and increasingly informs systemic therapy decisions (1-3). However, there has been controversy as to whether carriers of germline pathogenic variants (PVs) in *BRCA1* and *2* (*BRCA1/2*) or other cancer susceptibility genes benefit from some chemotherapy drugs more than others. The TNT trial reported greater efficacy of a platinum than a taxane for *BRCA1/2* PV carriers with metastatic hormone receptor (HR)–negative, HER2-negative (also known as triple-negative) breast cancer (TNBC) (4). Whereas the CALGB 40603 trial, first reported in 2015, showed higher pathologic complete response rates among TNBC patients treated with carboplatin (5), the subsequent GEPAR-SIXTO (published in 2017) (6) and INFORM (published in 2019) (7) trials demonstrated no improvement when a platinum was added to, or substituted for, standard chemotherapy for *BRCA1/2* PV carriers with early stage breast cancer. To our knowledge, no trial has demonstrated that *BRCA1/2* PV carriers benefit significantly from treatment intensification by adding an anthracycline to a taxane-based regimen or a larger number of chemotherapy agents (eg, ≥3 vs 2) to a regimen.

Reflecting the clinical uncertainty, practice guidelines have not advised more intensive chemotherapy regimens—defined by inclusion of more and/or different chemotherapy drugs—for *BRCA1/2* PV carriers vs noncarriers. However, in a prior population-based study, we found that receipt of any chemotherapy was higher among PV carriers than noncarriers, even with low-risk, HR-positive, HER2-negative disease for which guidelines do not advise chemotherapy (3,8). This raises the question of whether PV carriers may receive more intensive chemotherapy regimens than noncarriers—either more drugs, such as anthracyclines (9-11) or less standard breast cancer drugs such as platinums (8). Answering this question has implications for understanding how effectively genetic testing results are being implemented into oncology practice and for interpreting the cancer mortality differences by genetic results that have been reported in population-based studies (12-15).

We studied receipt of chemotherapy regimens in a population-based sample of women diagnosed with breast cancer in California or Georgia from 2013 to 2017 and reported to statewide Surveillance, Epidemiology and End Results (SEER) cancer registries, linked to their results of clinical germline genetic testing provided by 4 laboratories. Our primary hypothesis was that among women who underwent genetic testing and received first-course chemotherapy for breast cancer, *BRCA1/2* and other PV carriers were more likely than noncarriers to be treated with more intensive chemotherapy regimens, as defined by inclusion of more drugs, anthracyclines, and/or platinums.

## Methods

### Creation of Dataset and Analytic Cohort

We previously reported details of the Georgia and California SEER Genetic Testing Linkage Initiative (1,3,15-17). In brief, all women with breast cancer diagnosed at age 20 years or older from 2013 to 2017 and reported to the SEER registries of Georgia (Georgia Cancer Registry) and California (Los Angeles County, Greater Bay Area, and Greater California Registries) were linked with germline genetic testing results from 4 laboratories (Ambry Genetics, Aliso Viejo, CA, USA; Bioreference/GeneDx, Gaithersburg, MD, USA; Invitae, San Francisco, CA, USA; Myriad Genetics, Salt Lake City, UT, USA) that conducted the great majority of clinical testing in these regions over this period, according to surveys of clinicians and patients (16,18). Waivers of authorization and informed consent were approved by the institutional review boards of Georgia and California in accord with an assurance filed with and approved by the US Department of Health and Human Services, given collaboration with a third-party honest broker (Information Management Services, Inc, Rockport, MD, USA) to link the data and produce a de-identified research dataset including registry and testing laboratory information.

We recently published on breast cancer–specific mortality among women with stages I-IV breast cancer who received chemotherapy according to SEER records and linked to a genetic testing result from any of the 4 partnering laboratories (15). For the current study, we excluded patients with HER2-positive breast cancer because of definitive guidelines for HER2-directed systemic therapy regimens based on stage (8,19), such that chemotherapy is less likely to vary with PV status than for HER2-negative subtypes. We also excluded stage IV diagnoses to focus on curative-intent treatment. Given the labor-intensive process of collecting chemotherapy regimen data (described below), we could not include all eligible women from the prior mortality analysis (15) in the current study. To ensure sufficient sample size for the planned comparison of chemotherapy regimens between PV carriers and noncarriers, we included all eligible PV carriers from the prior mortality study (n = 1194) (15) and a random sample of women with other categories of genetic results to achieve the following distribution: 50% PV carriers and 50% with other genetic results (25% with variant of uncertain significance [VUS] only and 25% with negative genetic testing results; Supplementary Figure 1, available online).

### Testing Results From Laboratories

Partnering laboratories provided results of germline testing at the level of affected gene, including the interpretation sent to the ordering clinician: PV or likely PV (combined as PV for analysis), VUS, and benign or likely benign (combined for analysis as negative) (20). As previously described, results from all laboratories were pooled for anonymity; results were analyzed only for genes tested by at least 2 laboratories (n = 86 genes) (1,15,16).

### Chemotherapy Data Collection

SEER registries report a summary variable stating receipt or not of chemotherapy for first-course breast cancer treatment. The data reported to SEER registries come from facilities involved in the diagnosis and/or treatment of cancer patients in each registry’s coverage area. Included with these data are free text fields where the registrar is asked to enter details regarding the treatment rendered, including drug and regimen names or abbreviations (eg, “ddAC-T” for dose-dense doxorubicin and cyclophosphamide followed by taxol).

We developed an algorithm to automate review of these text fields and categorize drugs commonly used in adjuvant and/or neoadjuvant chemotherapy of stages I-III breast cancer into the following drug classes: anthracyclines (doxorubicin, epirubicin), cyclophosphamide, platinums (carboplatin, cisplatin), taxanes (docetaxel, paclitaxel), and other (all other drugs). The algorithm was initially developed and validated at the Georgia Cancer Registry. The validation dataset was created by registry staff manually coding drugs identified in the text fields. Algorithm validation occurred through an iterative process that compared random samples of the algorithm’s results against manually curated results obtained from registry staff. The algorithm was adjusted following each iteration until it consistently obtained a high level of coding accuracy. It was then applied to data from the California Cancer Registry, and the iterative process above was repeated using a similar validation dataset created by this registry. Once the algorithm performed consistently across Georgia and California registry datasets, it was run against the entire analytic cohort.

We grouped the drug classes that each patient received into a regimen approximating those recommended by guidelines of the National Comprehensive Cancer Network as follows: anthracycline and cyclophosphamide (AC); taxane and cyclophosphamide (TC); taxane and platinum (TP); anthracycline, cyclophosphamide, and taxane (ACT); and anthracycline, cyclophosphamide, taxane, and platinum (ACTP) (8). If a patient’s treatment did not meet criteria for any of these regimens, either because of absence of a required drug class or inclusion of another drug class, it was classified as “other.” All cases with regimens classified as other (n = 124) and a 10% subset of additional cases (n = 262) were reviewed and adjudicated by 2 board-certified medical oncologists (AWK and JCJ). Approximately 3% of cases did not have text specifically naming the agents administered and were excluded.

### Measures

Chemotherapy regimen intensiveness was defined separately for each of the 2 subtypes included: HR-positive, HER2-negative and TNBC. For each subtype, a more intensive regimen was defined as containing 1) more drugs than the minimum number in guideline-specified regimens and 2) an anthracycline and/or platinum (8). Thus, for HR-positive, HER2-negative disease, often treated with the 2-drug TC regimen (9,21), more intensive regimens were those including 3 or more drugs, at least 1 being an anthracycline. For TNBC, often treated with the 3-drug ACT regimen (10,22), more intensive regimens were those comprising 4 or more drugs, including both an anthracycline and a platinum. We coded the small number of patients in the “other” regimen category as having received less intensive treatment. A second measure of intensive therapy was based on chemotherapy type rather than drug number, specifically any use of a platinum (regardless of regimen received). Platinums are less standard in breast cancer treatment and have well-known toxicities such as neuropathy and thus arguably represent the most reliable indicator of a more aggressive treatment approach.

Additionally, SEER registries provided age at diagnosis, race and ethnicity, percent poverty at the census-tract level, tumor stage, grade, and subtype defined by expression of hormone receptors (estrogen and/or progesterone receptors) and HER2, and other first-course treatments (surgery and radiotherapy).

### Statistical Analysis

We described chemotherapy regimens by genetic results using the following mutually exclusive results subgroups: negative (no PV or VUS in any gene), VUS (in any gene, with no PVs), *BRCA1/2* PV, or other gene PV. We used separate multivariable models for each subtype to examine the association of receipt of more or less intensive chemotherapy regimens with genetic results groups, using covariates representing other factors that are known or likely to predict more or less intensive chemotherapy receipt. We used separate models for each subtype because of differences in the approach to treatment of HR-positive, HER2-negative vs TNBC. We also used multivariate models, separate for each subtype, to test whether PVs were associated with platinum receipt. Additional model covariates included patient sociodemographic factors (age at diagnosis, race and ethnicity, neighborhood poverty at the census-tract level), tumor clinical factors (stage, grade), year of diagnosis, and geographic site. All bivariate comparisons were tested using χ^2^ tests. All multivariable comparisons were tested using Wald F tests. All tests were 2 sided, using a statistical significance level of a *P* value less than .05.

## Results

### Patient Characteristics

Table 1 shows demographic and clinical characteristics of 2293 women who were diagnosed with stages I-III breast cancer from 2013 to 2017; reported to the California or Georgia SEER registries; linked to a genetic test result; had HR-positive, HER2-negative disease (n = 1451) or TNBC (n = 842); and received chemotherapy as first-course treatment. For both HR-positive, HER2-negative disease and TNBC, the most commonly received drug was cyclophosphamide, followed by taxanes, anthracyclines, platinums, and other agents. For both subtypes, anthracycline use declined with age (HR-positive, HER2-negative: 67% for ages younger than 50 years, 30% for ages 65 years and older; TNBC: 77% for ages younger than 50 years, 48% for ages 65 years and older) and increased with stage (HR-positive, HER2-negative: stage I, 27%; stage III, 76%; TNBC: stage I, 53%; stage III, 84%).

**Table 1.** Patient characteristics and chemotherapy drug classes received, by breast cancer subtype

Patient characteristics and treatments	Hormone receptor-positive, HER2-negative						Triple-negative					
	No.	Anthracycline, %	Platinum, %	Taxane, %	Cyclophosphamide, %	Other, %	No.	Anthracycline, %	Platinum, %	Taxane, %	Cyclophosphamide, %	Other, %
Genetic test result												
Negative	377	55	6	93	94	7	186	68	22	92	90	7
*BRCA1/2*	358	67	14	89	90	6	369	72	33	88	85	5
Other PV	335	51	4	89	93	7	132	66	30	86	83	8
VUS only	381	52	6	91	92	6	155	75	23	89	90	9
Age at diagnosis, y												
Younger than 50	815	67	8	91	94	6	408	77	32	89	87	7
50-64	478	46	6	90	91	8	335	69	25	89	88	7
65 and older	158	30	8	90	88	6	99	48	34	85	81	5
Race and ethnicity^a^												
Asian/Pacific Islander	159	50	9	92	91	6	69	61	29	91	77	7
Black	225	63	11	90	92	9	191	75	25	87	92	10
Hispanic	233	65	8	87	92	8	152	74	27	86	86	3
Non-Hispanic White	829	53	6	91	93	5	422	69	31	90	86	7
Stage												
1	292	27	7	91	91	2	257	53	17	90	89	4
2	777	57	7	89	92	5	456	77	33	88	85	6
3	382	76	9	92	94	12	129	84	35	87	88	14
Grade^a^												
1	114	45	4	90	96	7	7	57	43	86	86	0
2	618	53	5	90	92	5	91	64	25	84	85	10
3	690	61	11	91	92	7	714	71	28	90	87	7
Surgery												
Lumpectomy	437	44	7	90	93	5	263	67	20	88	90	5
Unilateral mastectomy	319	57	4	91	95	9	139	71	23	87	90	12
Bilateral mastectomy	486	66	9	92	91	5	305	74	34	90	86	6
Mastectomy, NOS	153	49	10	89	88	4	89	71	39	91	83	8
No surgery	56	80	11	77	89	14	46	70	37	85	72	7
Radiation therapy												
No	596	49	8	86	88	6	455	66	27	86	83	6
Yes	855	61	7	93	95	7	387	76	30	92	91	8
Geographic site												
California	894	54	9	89	90	6	491	68	31	86	81	7
Georgia	557	60	6	93	95	7	351	75	25	93	94	7
Neighborhood poverty												
<10%	658	52	8	93	93	5	343	69	30	90	86	4
10%-19%	455	57	7	90	91	7	291	70	27	89	87	9
≥20%	338	62	7	86	93	7	207	74	27	86	88	8
Year of diagnosis												
2013	170	55	7	90	91	5	93	65	27	88	86	7
2014	322	56	8	90	93	6	185	68	28	89	87	7
2015	327	57	7	90	92	5	204	66	31	89	85	5
2016	319	56	9	91	89	7	183	70	27	91	86	7
2017	313	55	7	91	95	7	176	77	26	86	90	9

a Missing and other or unknown results are not shown: for race and ethnicity, n = 13 other or unknown; for grade, n = 59 missing; for neighborhood poverty, n = 1 missing. *BRCA1/2* = *BRCA1* and/or *BRCA2*; NOS = not otherwise specified; PV = pathogenic variant; VUS = variant of uncertain significance.

### Chemotherapy Regimens Received by Genetic Results

**Figure 1.**
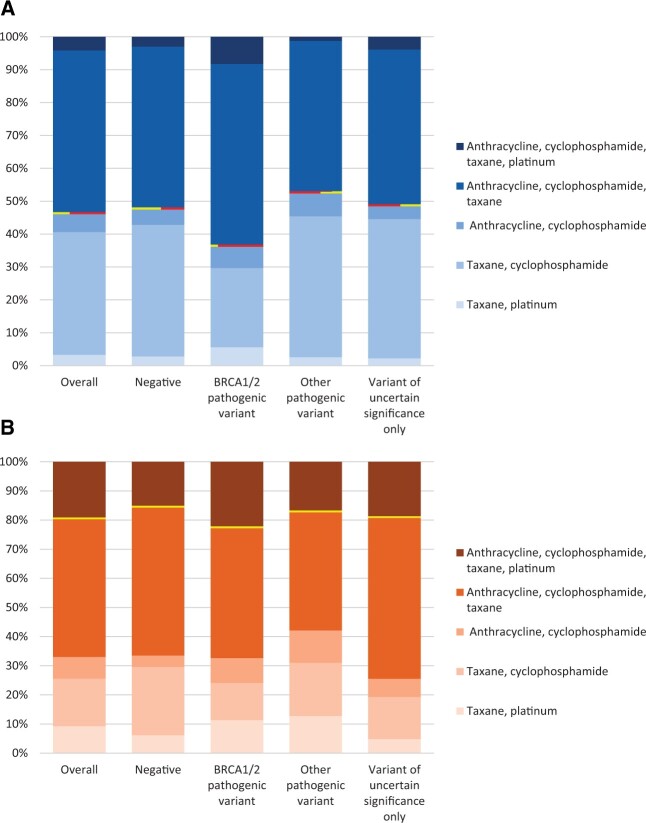
Receipt of chemotherapy regimens containing anthracyclines, cyclophosphamide, platinums, or taxanes by results of germline genetic testing by women with **(A)** hormone receptor-positive, HER2-negative and (**B)** triple-negative breast cancer. The red/yellow horizontal line in each figure separates more intensive from less intensive chemotherapy regimens for each subtype. Patients who received another regimen, classified as “other,” were excluded because of small numbers (n = 124, 5%). *BRCA1/2* = *BRCA1* and/or *BRCA2*

Figure 1 shows chemotherapy regimens received by genetic results, excluding those classified to the regimen “other” (n = 124, 5.4%), and designating the more intensive regimen(s) for each subtype above a dividing line. Among women with HR-positive, HER2-negative disease, the distribution of treatment regimens for *BRCA1/2* PV carriers was ACT (55.3%), TC (24.3%), ACTP (8.3%), AC (6.5%), and TP (5.6%); for women testing negative, it was ACT (49.2%), TC (40.3%), AC (4.7%), ACTP (3.0%), and TP (2.8%). Among women with HR-positive, HER2-negative disease, more intensive regimens (ACT plus ACTP) were received by 63.6% of *BRCA1/2* PV carriers and 52.2% of women testing negative. Among women with TNBC, the distribution of treatment regimens for *BRCA1/2* PV carriers was ACT (44.9%), ACTP (22.3%), TC (12.9%), TP (11.4%), and AC (8.6%); for women testing negative, it was ACT (51.1%), TC (23.6%), ACTP (15.2%), TP (6.2%), and AC (3.9%). Among women with TNBC, a more intensive regimen (ACTP) was received by 22.3% of *BRCA1/2* PV carriers and 15.2% of women testing negative. For both subtypes, the distribution of regimens received by women with another gene PV or VUS-only results were similar to those of women testing negative.

### Multivariable Models of More Intensive Regimen Receipt

Table 2 shows multivariable model results by subtype for receipt of a more intensive chemotherapy regimen. Among women with HR-positive, HER2-negative disease, receipt of a more intensive regimen varied significantly by genetic result (*P* = .02): the odds ratio (OR) for *BRCA1/2* PV carriers was 1.35 (95% confidence interval [CI] = 0.96 to 1.90; *P* = .09) vs 0.8 (95% CI = 0.57 to 1.12; *P* = .19) for other gene PV carriers and 0.91 (95% CI = 0.66 to 1.25; *P* = .55) for patients with VUS-only results. Among women with TNBC, receipt of a more intensive chemotherapy regimen varied somewhat by genetic result but did not reach statistical significance (*BRCA1/2* PV carriers OR = 1.66, 95% CI = 0.97 to 2.82; *P* = .27). For women with HR-positive, HER2-negative disease, other features significantly associated with receipt of a more intensive regimen included younger age (OR = 0.49, 95% CI = 0.38 to 0.63; *P* < .001), higher stage (OR = 9.56, 95% CI = 6.56 to 13.93; *P* < .001), and higher grade (OR = 0.56, 95% CI = 0.35 to 0.88 for grade 1 vs grade 3; *P* = .01); for women with TNBC, only higher stage was associated with a more intensive regimen (OR = 4.59, 95% CI = 2.41 to 8.75; *P* < .001). Neither race and ethnicity nor neighborhood poverty was associated with any difference in the chemotherapy regimen received. In a sensitivity analysis excluding those with regimens classified as “other” and coded as “less intensive” for this model (n = 124, 5%), results were not meaningfully different (Supplementary Table 1, available online).

**Table 2.** Multivariable models of receipt of a more intensive chemotherapy regimen, by breast cancer subtype^a^

Patient characteristics	HR-positive, HER2-negative		Triple-negative	
	Odds ratio (95% CI)	*P*	Odds ratio (95% CI)	*P*
Genetic test result		.02		.27
Negative	Referent		Referent	
*BRCA1/2*	1.35 (0.96 to 1.90)		1.66 (0.98 to 2.83)	
Other PV	0.8 (0.57 to 1.12)		1.23 (0.64 to 2.38)	
VUS only	0.91 (0.66 to 1.25)		1.25 (0.67 to 2.32)	
Age at diagnosis, y		<.001		.07
Younger than 50	Referent		Referent	
50-64	0.49 (0.38 to 0.63)		0.84 (0.56 to 1.26)	
65 and older	0.26 (0.17 to 0.40)		0.40 (0.18 to 0.88)	
Race and ethnicity		.82		.40
Asian/Pacific Islander	0.86 (0.58 to 1.27)		0.50 (0.21 to 1.18)	
Black	0.95 (0.66 to 1.36)		0.88 (0.52 to 1.46)	
Hispanic	1.06 (0.74 to 1.50)		0.77 (0.45 to 1.33)	
Non-Hispanic White	Referent		Referent	
Stage		<.001		<.001
1	Referent		Referent	
2	3.46 (2.5 to 4.78)		3.70 (2.16 to 6.36)	
3	9.56 (6.56 to 13.9)		4.59 (2.41 to 8.75)	
Grade		.007		.88
1^b^	0.56 (0.35 to 0.88)		—	
2	0.73 (0.57 to 0.93)		1.05 (0.58 to 1.91)	
3	Referent		Referent	
Geographic site		<.001		.12
California	Referent		Referent	
Georgia	0.60 (0.46 to 0.79)		0.69 (0.44 to 1.10)	
Neighborhood poverty		.76		.67
<10%	Referent		Referent	
10%-19%	0.91 (0.69 to 1.20)		0.82 (0.52 to 1.27)	
≥20%	1.01 (0.74 to 1.38)		0.91 (0.56 to 1.48)	
Year of diagnosis, per year	1.03 (0.90 to 1.18)	.70	0.84 (0.67 to 1.05)	.12

^a^ More intensive chemotherapy regimens: for hormone receptor-positive, HER2-negative: ≥3 drugs, including an anthracycline; for triple-negative: ≥4 drugs, including an anthracycline and a platinum. — = no results available; *BRCA1/2* = *BRCA1* and/or *BRCA2*; CI = confidence interval; PV = pathogenic variant; VUS = variant of uncertain significance.

b Grade 1 was excluded from the triple-negative model because of small numbers (n = 7).

### Multivariable Model of Platinum Receipt

Table 3 shows multivariable models by subtype for receipt of a platinum. Among women with HR-positive, HER2-negative disease, receipt of a platinum varied significantly by genetic result (*P* < .001); compared with women testing negative, *BRCA1/2* PV carriers had twice the odds of receiving a platinum (OR = 2.44, 95% CI = 1.36 to 4.38; *P* = .003). Among women with TNBC, again the direction of effects was similar to those for women with HR-positive, HER2-negative disease but did not meet statistical significance (*P* = .08). For women with HR-positive, HER2-negative disease, other factors associated with platinum receipt included higher grade (OR = 0.44, 95% CI = 0.27 to 0.70 for grade 2 vs grade 3; *P* < .001) and Black race (OR = 2.21, 95% CI = 1.20 to 4.06; *P* = .01). For women with TNBC, higher stage was associated with more platinum receipt (OR = 2.93, 95% CI = 1.75 to 4.91; *P* < .001), whereas more recent diagnosis was associated with less platinum receipt (OR per year = 0.8, 95% CI = 0.66 to 0.97; *P* = .02). Neighborhood poverty was not associated with platinum receipt.

**Table 3.** Multivariable models of receipt of a platinum, by breast cancer subtype

Patient characteristics	HR-positive, HER2-negative		Triple-negative	
	Odds ratio (95% CI)	*P*	Odds ratio (95% CI)	*P*
Genetic test result		<.001		.08
Negative	Referent		Referent	
*BRCA1/2*	2.44 (1.36 to 4.38)		1.60 (1.03 to 2.51)	
Other PV	0.66 (0.32 to 1.38)		1.50 (0.87 to 2.56)	
VUS only	1.13 (0.60 to 2.14)		0.99 (0.57 to 1.70)	
Age at diagnosis, y		.23		.14
Younger than 50	Referent		Referent	
50-64	0.93 (0.57 to 1.52)		0.77 (0.54 to 1.10)	
65 and older	1.71 (0.87 to 3.35)		0.60 (0.34 to 1.07)	
Race and ethnicity		.06		.15
Asian/Pacific Islander	1.14 (0.59 to 2.22)		0.70 (0.38 to 1.29)	
Black	2.21 (1.20 to 4.06)		0.81 (0.51 to 1.28)	
Hispanic	0.89 (0.48 to 1.67)		0.60 (0.37 to 0.96)	
Non-Hispanic White	Referent		Referent	
Stage		.16		<.001
1	Referent		Referent	
2	1.15 (0.65 to 2.02)		2.54 (1.70 to 3.80)	
3	1.68 (0.91 to 3.11)		2.93 (1.75 to 4.91)	
Grade		.001		.98
1^a^	0.41 (0.14 to 1.17)		—	
2	0.44 (0.27 to 0.70)		0.99 (0.60 to 1.64)	
3	Referent		Referent	
Geographic site		.02		.20
California	Referent		Referent	
Georgia	1.87 (1.09 to 3.19)		1.29 (0.87 to 1.92)	
Neighborhood poverty		.29		.67
<10%	Referent		Referent	
10%-19%	0.68 (0.41 to 1.13)		0.87 (0.60 to 1.27)	
≥20%	0.75 (0.43 to 1.31)		0.84 (0.55 to 1.29)	
Year of diagnosis, per year		.51		.02
	0.92 (0.73 to 1.17)	.39	0.80 (0.66 to 0.97)	

^a^ Grade 1 was excluded from the triple-negative model because of small numbers (n = 7). — = no results available; CI = confidence interval; *BRCA1/2* = *BRCA1* and/or *BRCA2*; PV = pathogenic variant; VUS = variant of uncertain significance.

## Discussion

This is the first study to characterize breast cancer chemotherapy regimens received by women with germline PVs in community practice. We observed differences in receipt of a more intensive regimen among PV carriers vs noncarriers: *BRCA1/2* PV carriers with HR-positive, HER2-negative breast cancer received more intensive regimens, particularly as defined by platinum use. Chemotherapy regimens did not vary between women with PVs in other genes, VUS-only and negative genetic results, or by neighborhood poverty. These findings inform our understanding of treatment quality after genetic testing, as indicated by adherence or not to evidence-based guidelines. They also guide interpretation of results of prior studies that compared breast cancer–specific mortality between *BRCA1/2* PV carriers and noncarriers (12-15,23).

Genetic testing has rapidly entered oncology practice, with major implications for targeted screening, risk-reducing surgery, and systemic therapy. The recent US Food and Drug Administration approval of drugs that target genetic results, such as poly(ADP-ribose) polymerase inhibitors for *BRCA1/2* PV carriers with breast and other cancers (2,12,24-27) and immune checkpoint inhibitors for any advanced solid tumor with mismatch repair deficiency (28,29), offers strong evidence of benefit from genetic testing. However, there is also evidence of mismatch between genetic results and therapies received, for example, unwarranted recommendation of risk-reducing salpingo-oophorectomy to patients without an elevated risk of ovarian cancer in the PROMPT study (30) and our prior work suggesting underuse of radiotherapy and overuse of chemotherapy among *BRCA1/2* PV carriers (3). The current results offer more evidence that treatment selection may deviate from clinical guidelines for PV carriers and emphasize the need to monitor how genetic results are managed in oncology practice.

Practice guidelines do not advise that platinum agents should be used for most patients with early stage, HER2-negative breast cancer (8); thus, we were surprised to find twofold greater odds of platinum receipt by *BRCA1/2* PV carriers vs noncarriers with HR-positive, HER2-negative disease. It was also unexpected to find that receipt of more intensive, anthracycline-containing regimens varied by genetic results in HR-positive, HER2-negative disease, particularly given declining anthracycline use among US breast cancer patients overall in a MarketScan claims analysis (9); however, more platinum receipt was likely the primary driver of this result. Notably, we previously found no lower breast cancer–specific mortality among *BRCA1/2* PV carriers vs noncarriers with HR-positive, HER2-negative disease in an analysis that included all women in the current study (15); thus, there is no evidence of mortality reduction from the more intensive regimens that *BRCA1/2* PV carriers received for HR-positive, HER2-negative breast cancer. Taken together, these results suggest overtreatment of HR-positive, HER2-negative disease in *BRCA1* and *2* PV carriers, which may expose patients unnecessarily to toxicities including neuropathy (platinums), cardiomyopathy, and secondary hematologic malignancies (anthracyclines). The twofold higher odds of platinum receipt by Black patients with HR-positive, HER2-negative disease also warrants investigation as a potential health disparity.

Among women with TNBC, no significant difference in platinum receipt was observed by genetic results. This finding may reflect the smaller sample size than with HR-positive, HER2-negative disease and may also reflect the relatively high use of intensive, platinum-containing regimens among all TNBC patients. The present results—that *BRCA1/2* PV carriers do not receive substantially different chemotherapy regimens than noncarriers for TNBC—further inform interpretation of our prior research showing lower TNBC-specific mortality among these *BRCA1/2* PV carriers vs noncarriers (15). Because they are treated similarly to noncarriers, any lower TNBC mortality among PV carriers likely reflects a general chemosensitivity consistent with their underlying DNA repair defect rather than an effect of differential treatment. This finding is concordant with results of the INFORM trial, which showed equivalent response of *BRCA1/2*-associated breast cancers to several different chemotherapy agents (7): taken together, they suggest that comparison of different chemotherapy drugs should not be a major focus of future clinical trials in *BRCA1/2*-associated TNBC.

Aspects of this study must be considered in interpreting its results. We ascertained chemotherapy regimen details through review of SEER fields containing free text entered by registrars; this methodology has been used in SEER-based studies of breast cancer treatment and the impact of COVID-19 on cancer care (31,32) but has otherwise not been widely validated. Patients were diagnosed with breast cancer from 2013 to 2017, when more recently approved targeted therapies including poly(ADP-ribose) polymerase and immune checkpoint inhibitors were not yet available (2,33). Furthermore, chemotherapy practice patterns may have changed since 2017, after publication of the CALGB 40603 and GEPAR-SIXTO trials focused on carboplatin (5,6) and the ABC and PlanB trials focused on anthracycline vs nonanthracycline-based regimens (10,34). Notably, however, we observed no decline over time in platinum or intensive chemotherapy regimen receipt for patients with HR-positive, HER2-negative disease. We did not have information on the degree of positivity of tumor estrogen and progesterone receptor expression or on results of the 21-gene recurrence score assay, if performed. As noted above, women who underwent genetic testing may have done so because of a perceived worse prognosis and thus might not reflect all breast cancer patients; however, they do represent women tested in community practice in 2 large, diverse states. A considerable strength is this study’s use of population-based, statewide SEER registries linked directly to clinical genetic results from testing laboratories; this minimizes selection bias and offers broad generalizability for US breast cancer patients.

We found that among early stage, HR-positive, HER2-negative breast cancer patients, *BRCA1*/*2* PV carriers receive more intensive, platinum-based chemotherapy regimens than other patients, for which there is neither evidence of benefit nor guideline recommendation (8). Long-term follow-up of guideline adherence after genetic testing, and of cancer recurrence and mortality among PV carriers, is essential, particularly in the context of emerging genetically targeted adjuvant therapies (2,33).

## Funding

Research reported in this publication was supported by the National Cancer Institute (NCI) of the National Institutes of Health under award numbers P01 CA163233 to the University of Michigan and R01 CA225697 to Stanford University. The collection of cancer incidence data in Georgia was supported by contract HHSN261201800003I, Task Order HHSN26100001 from the NCI and cooperative agreement 5NU58DP006352-03-00 from the CDC. The collection of cancer incidence data used in this study was supported by the California Department of Public Health pursuant to California Health and Safety Code Section 103885; Centers for Disease Control and Prevention’s (CDC) National Program of Cancer Registries, under cooperative agreement 5NU58DP006344; the National Cancer Institute’s Surveillance, Epidemiology and End Results Program under contract HHSN261201800032I awarded to the University of California, San Francisco, contract HHSN261201800015I awarded to the University of Southern California, and contract HHSN261201800009I awarded to the Public Health Institute, Cancer Registry of Greater California.

## Notes

**Role of the** **funders:** The funders had no role in the design of the study; the collection, analysis and interpretation of the data; the writing of the manuscript; and the decision to submit the manuscript for publication.

**Disclosures:** Research funding to her institution for an unrelated project was provided to Allison Kurian, MD, MSc, by Myriad Genetics (2017-2019). The authors have no conflicts of interest to declare.

**Author** **contributions:** Conceptualization: AK, SK, KW; Formal analysis: PA, TH; Data curation: PA; Resources: KW, AH, SG; Writing—original draft: AK, SK, KW; Writing—review and editing: AK, PA, AH, SG, JCJ, TH, SK, KW; Funding acquisition: AK, SK

**Prior** **presentations:** No portion of this manuscript has been published previously. Preliminary results in partial form were presented at the American Society of Clinical Oncology Annual Meeting, Chicago, IL, June 6, 2022.

**Disclaimer:** The ideas and opinions expressed herein are those of the authors and do not necessarily reflect the opinions of the State of California, Department of Public Health, the National Cancer Institute, and the Centers for Disease Control and Prevention or their Contractors and Subcontractors.

## Supplementary Material

pkac045_Supplementary_Data

## Data Availability

The data underlying this article cannot be shared publicly at this time because of agreements with participating testing laboratories.
