# Supplementary material for: Chemotherapy Regimens Received by Women With BRCA1/2 Pathogenic Variants for Early Stage Breast Cancer Treatment
Source: JNCI Cancer Spectr. 2022 Jun 20;6(4):pkac045. doi: 10.1093/jncics/pkac045 (PMC9305849; doi:10.1093/jncics/pkac045)
Supplement: pkac045_Supplementary_Data [file pkac045_supplementary_data.pdf]

SUPPLEMENTARY MATERIALS

22,495 women[a]:
- Diagnosed with Stages I-IV breast cancer in 2013-2017
- Reported to Georgia or California Surveillance, Epidemiology and End Results (SEER) registries
- Received chemotherapy as first-course treatment according to SEER records
- Linked to clinical germline genetic testing results from participating laboratories

7,843 women excluded for:
- Stage IV
- HER2-posititve subtype

14,652 women remaining:
- Diagnosed with Stages I-III breast cancer in 2013-2017
- Reported to Georgia or California SEER registries
- Received chemotherapy as first-course treatment according to SEER records
- Linked to clinical germline genetic testing results from participating laboratories
- Hormone receptor-positive (HR-positive)/HER2-negative or triple-negative subtype

Included in analytic cohort: 50% with pathogenic variants, 50% with other testing results (2,293)
- All available pathogenic variant carriers (1,194): 727 in *BRCA1/2*, 467 in other genes
- Randomly selected women with other results (1,099): 563 negative, 536 uncertain significance

Supplementary Figure 1. Flow of Patients into the Analytical Cohort. [a]These 22,495 women were included in our prior study of breast cancer-specific mortality according to germline genetic testing results: Kurian AW, Abrahamse P, Bondarenko I, et al. Association of genetic testing results with mortality among women with breast cancer or ovarian cancer. Journal of the National Cancer Institute 2022 Feb 7;114(2):245-253

**Supplementary Table 1.** Multivariable models of receipt of a more intensive chemotherapy regimen[a], by breast cancer subtype, excluding "other" regimens[b]				
	Hormone receptor-positive, HER2-negative		Triple-negative	
	Odds Ratio (95% CI)	p	Odds Ratio (95% CI)	p
Genetic Test Result		.02		.36
Negative	Reference		Reference	
BRCA1/2	1.479 (1.039 to 2.105)		1.609 (0.944 to 2.741)	
Other PV	0.858 (0.608 to 1.21)		1.255 (0.649 to 2.426)	
VUS only	0.957 (0.689 to 1.331)		1.290 (0.691 to 2.411)	
Age at Diagnosis		<.001		.06
<50 years	Reference		Reference	
50-64 years	0.515 (0.395 to 0.672)		0.824 (0.546 to 1.244)	
≥65 years	0.269 (0.174 to 0.414)		0.387 (0.174 to 0.864)	
Race and Ethnicity		.69		.35
Asian/Pacific Islander	0.841 (0.566 to 1.251)		0.485 (0.205 to 1.147)	
Black	1.014 (0.697 to 1.475)		0.868 (0.516 to 1.461)	
Hispanic	1.130 (0.783 to 1.632)		0.749 (0.430 to 1.303)	
Non-Hispanic White	Reference		Reference	
Stage		<.001		<.001
1	Reference		Reference	
2	3.605 (2.591 to 5.015)		3.758 (2.183 to 6.470)	
3	10.245 (6.941 to 15.122)		4.699 (2.456 to 8.992)	
Grade		.008		.79
1[c]	0.543 (0.340 to 0.866)		-	
2	0.727 (0.563 to 0.939)		1.084 (0.591 to 1.988)	
3	Reference		Reference	
Geographic Site		<.001		.22
California	Reference		Reference	
Georgia	0.604 (0.454 to 0.803)		0.749 (0.471 to 1.190)	
Neighborhood Poverty		.76		.71
<10% poverty	Reference		Reference	
10%-19% poverty	0.928 (0.697 to 1.235)		0.836 (0.535 to 1.307)	
≥20% poverty	1.046 (0.757 to 1.446)		0.969 (0.593 to 1.583)	
Year of Diagnosis (per year)		.99		.06
	0.999 (0.869 to 1.148)		0.809 (0.647 to 1.011)	

Abbreviations: CI, confidence interval; *BRCA1/2*, *BRCA1* and/or *BRCA2*; PV, pathogenic variant; VUS, variant of uncertain significance

[a]More intensive chemotherapy regimens: for hormone receptor-positive, HER2-negative: ≥3 drugs, including an anthracycline; for triple-negative: ≥4 drugs, including an anthracycline and a platinum

[b]The drug classes that each patient received were grouped into regimens approximating those recommended by guidelines of the National Comprehensive Cancer Network (http://www.nccn.org), as follows: Anthracycline/Cyclophosphamide (AC), Taxane/Cyclophosphamide (TC), Taxane/Platinum (TP), Anthracycline/Cyclophosphamide/Taxane (ACT), and Anthracycline/Cyclophosphamide/Taxane/Platinum (ACTP). If a patient's treatment did not meet criteria for any of these regimens, either due to absence of a required drug class or inclusion of another drug class, it was classified as "other".

[c]Grade 1 was excluded from the triple-negative model due to small numbers (n=7)
